# The SH3PXD2A-HTRA1 fusion transcript is extremely rare in Norwegian sporadic vestibular schwannoma patients

**DOI:** 10.1007/s11060-021-03796-6

**Published:** 2021-07-02

**Authors:** Peter Taule-Sivertsen, Ove Bruland, Aril Løge Håvik, Eirik Bratland, Morten Lund-Johansen, Per Morten Knappskog

**Affiliations:** 1 Department of Clinical Science, University of Bergen, Bergen, Norway; 2 Department of Medical Genetics, Haukeland University Hospital, Bergen, Norway; 3 Department of Neurosurgery, Haukeland University Hospital, Bergen, Norway

**Keywords:** Vestibular schwannoma, Gene fusion, Tumorigenesis, Driver mutation, Neurosurgery, Genetics

## Abstract

**Introduction:**

Vestibular schwannoma (VS) is a benign intracranial tumor in which the underlying genetics is largely uncertain, apart from mutations in the tumor suppressor gene *NF2*. Alternative tumorigenic mechanisms have been proposed, including a recurrent in-frame fusion transcript of the *HTRA1* and *SH3PXD2A* genes. The gene product of the *SH3PXD2A-HTRA1* fusion has been shown to promote proliferation, invasion and resistance to cell death in vitro and tumor growth in vivo. The aim of this study was to replicate the findings and to investigate the frequency of this fusion gene in another cohort of vestibular schwannoma patients.

**Methods:**

The *SH3PXD2A-HTRA1* transcript was synthesized in vitro using PCR and used as a positive control to assess the sensitivity of a real-time PCR assay. This real-time PCR assay was used to search for the presence of the fusion transcript in 121 Norwegian sporadic VS patients.

**Results:**

The real-time PCR assay showed a high sensitivity and was able to detect as low as ~ 5 copies of the fusion transcript. Out of the 121 investigated tumors, only 1 harbored the *SH3PXD2A-HTRA1* fusion.

**Conclusion:**

Even though the *SH3PXD2A-HTRA1* fusion has been shown to be a driver of tumorigenesis, our results suggest that it is a rare event in our VS patients. Further investigation is warranted in order to elucidate whether our results represent an extreme, and if the fusion is present also in other neoplasms.

**Supplementary Information:**

The online version contains supplementary material available at 10.1007/s11060-021-03796-6.

## Introduction

Vestibular schwannoma (VS) is a benign intracranial tumor originating from the myelin producing Schwann cells, which in most cases arise sporadically. The reported annual incidence rate of these tumors is rising, and ranges from 11 to 30.7 per million [1, 2]. Because the tumor mostly affects the vestibulocochlear nerve, early symptoms of VS are usually hearing loss and tinnitus, while later symptoms of large tumors include nausea and imbalance [3]. VS can therefore greatly reduce the patient’s quality of life and can even be a cause of death, due to obstructive hydrocephalus and increased intracranial pressure. The tumorigenesis of VS is largely associated with loss-of-function mutations in the *NF2* gene, which encodes the tumor suppressor protein *Merlin*. However, impaired function of Merlin is also found in VSs that do not harbor mutations in that particular gene, suggesting that additional members of the NF2/Hippo pathway could be involved. The proportion of sporadic VSs that *do* contain *NF2* mutations ranges from 15 to 100% [4]. The lower proportions reported could be due to many VSs having a high inflammatory component [5], poor sensitivity of techniques, or other drivers of VS tumorigenesis that are yet to be disclosed. This has caused further investigation into the pathogenesis of VS and the revelation of other tumorigenic factors, e.g., the cancer-related axonal guidance pathway, mutations in the genes *CDC27* and *USP8* and downregulation of *CAV1* [6, 7]. Several of the other mutations found in VS can be linked to *NF2* function. Another gene of interest is *HTRA1*, encoding a serine protease and reported as a possible oncogene [8].

Translocations and their corresponding gene fusions have been established as important contributing tumorigenic factors in various benign and malignant neoplasms [9]. In 2016, Agnihotri et al. published the finding of a frequently recurrent in-frame fusion between the *SH3PXD2A* and *HTRA1* genes in 12/125 (10%) of their schwannoma patients, including 7/64 (11%) of their VS patients [10]. They first discovered the fusion in RNA-sequencing data in tumors from 5/41 patients. The fusion junction was identical in these 5 tumors, and RT-PCR was used to validate the findings in an additional 7/84 samples. This fusion was also found by Aaron et al., who found it in 1/14 VS patients subjected to RNA sequencing [11]. Only 2/7 of the fusion-positive VS patients reported by Agnihotri et al. had no *NF2* mutations, thus challenging the prospect of the fusion being an alternative tumorigenic pathway. However, the fusion gene product was shown to induce tumorigenic properties in vitro and in vivo through elevated phosphorylation of *ERK*, and pharmacologic inhibition of the *MEK-ERK* pathway reversed this effect. This finding has potential implications for the management of VS. Hence, we investigated the frequency of this genetic aberration in VS tissue isolated from Norwegian VS patients.

## Methods

### Patient material

Tissue biopsy samples were collected from 121 Norwegian patients operated for VS at the Department of Neurosurgery during the years 2013–2020. All patients signed a written consent before tissue harvesting, and the study was approved by the Regional Ethical committee for medical research in Western Norway (2013/374). RNA was isolated using QIAGEN’s *RNeasy Mini Kit.* Quality and concentration of the extracted RNA were checked with Agilent’s 2100 Bioanalyzer Instrument and Thermo Fisher’s *Nanodrop,* and reverse transcription of RNA to cDNA was done using Invitrogen’s *SuperScript *VILO cDNA Synthesis Kit. This cDNA was used to amplify the *SH3PXD2A* and *HTRA1* genes.

### Construction of the SH3PXD2A-HTRA1 fusion transcript

A PCR product representing the fusion between *HTRA1* and *SH3PXD2A* was generated by amplification of the two regions in question for the respective genes. These contained overlapping sequences representing the region containing the fusion added to the 5’ end of the PCR primers. This sequence enabled the two products to anneal to each other in a separate subsequent PCR reaction, creating a template for amplification of the fusion product (see supplementary material for primer details and PCR setup). 2 µl of the product from each of the first PCR reactions were mixed and used as DNA template in this new PCR reaction, yielding the fusion transcript (see supplementary material).

**Fig. 1:**
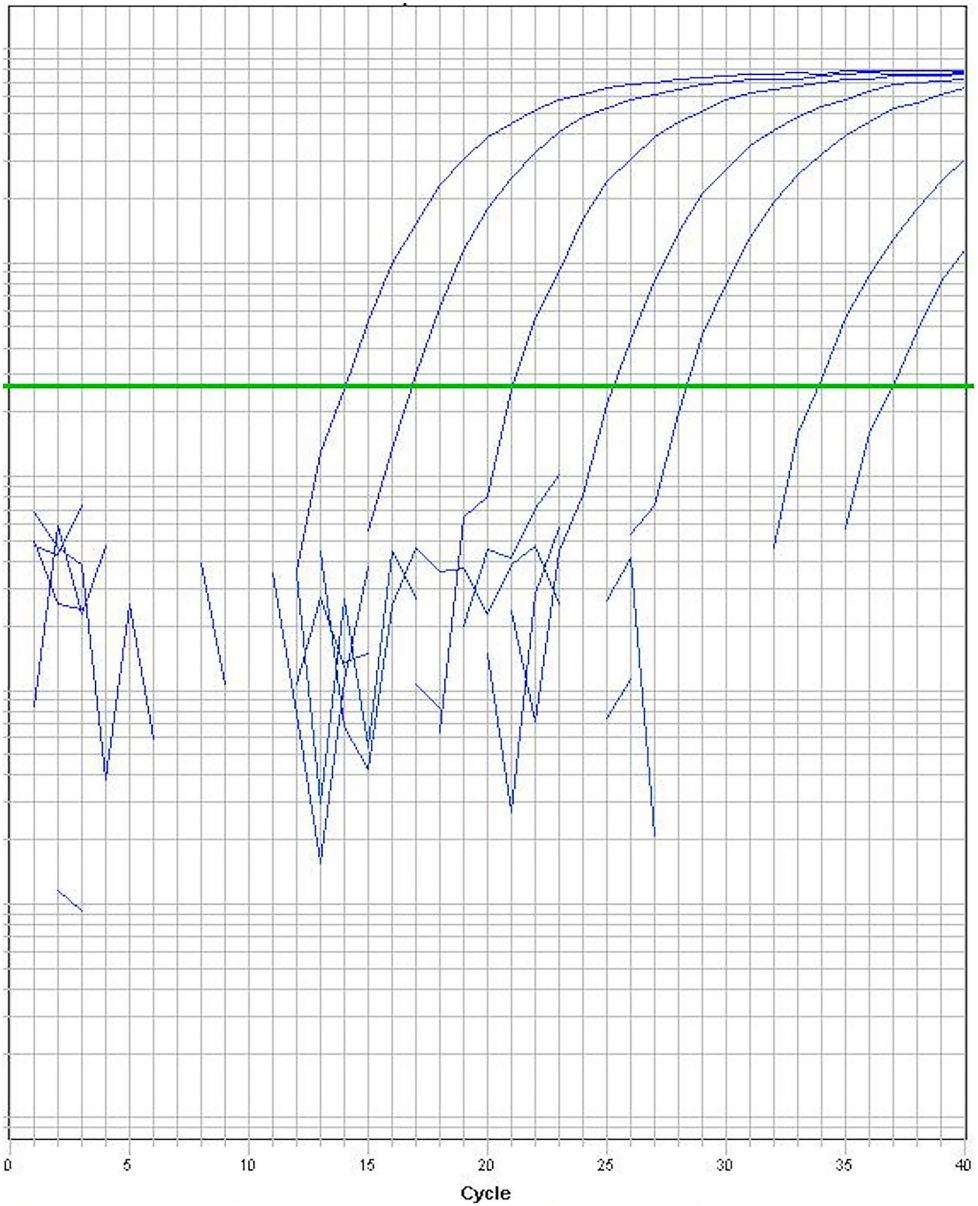
Detection of the fusion gene using real-time PCR. The serial dilution (1:10) is illustrated, starting with ~ 4.7 million copies. The last amplification curve (Ct: 36.9) to cross the threshold line contained ~ 5 copies of the fusion gene

The PCR product containing the fusion transcript was then cloned using Invitrogen’s *pcDNA 3.1/V5-His TOPO TA* vector *(45-0005)* using TOP10 chemically competent *E.coli* cells. The plasmids were isolated from the bacterial cells using QIAGEN’s *QIAprep Spin Miniprep Kit*. The plasmids were Sanger sequenced using the *BigDye Terminator v3.1 Cycle Sequencing Kit,* and *ABI3730 DNA Analyzer (*Thermo Fisher). *SnapGene Viewer* was used to analyze the results and to ensure that the correct transcript had been synthesized. The plasmids were then diluted to create a dilution series. The plasmid concentrations were measured using *Nanodrop*, and the concentration of the selected plasmid sample was 291 ng/µl. Initially, 1 µl of this plasmid sample was added to 10 ml MQ. Then, 1 µl of this diluted sample was added to 9 µl MQ. This process was repeated seven times, yielding eight solutions with different plasmid concentrations (Fig. 1). This serial dilution was included as a positive control in every real-time PCR reaction.

### Real-time PCR assay for detection of the SH3PXD2A-HTRA1 fusion transcript in patient samples

For the real-time PCR assay we used the same probe and primers as described in the original report. Actin-β was included as an internal amplification control. The reaction volume was 10 µl, and it contained 0.8 µl of the fusion gene primers/probe, 0.2 µl of actin-β gene primers/probe, 1 µl cDNA, 5 µl *TaqMan Universal PCR Master Mix* (Thermo Fisher Scientific) and 3 µl MQ.

### Whole-genome sequencing (WGS) and RNAseq

WGS and RNAseq (whole-transcriptome sequencing) data were available for the fusion-positive tumor from a previous study [12]. WGS had mean read depth of 30x, RNA-seq was done to a total of 273 million reads. Both were paired-end short reads. The suspected fusion breakage points were manually inspected in Integrative Genomics Viewer [13].

## Results

### Validity and sensitivity of the real-time PCR assay

The slopes of the amplification curves of the diluted plasmid indicate an efficient amplification of the fusion gene. The concentration of the initial plasmid solution and the molecular weight of the fusion-containing plasmid were used to approximate the number of copies of the fusion gene in each solution. The starting solution was estimated to contain ~ 4.7 million copies of the fusion gene per µl, meaning that the most diluted solution (1/100,000,000,000) should contain an estimated 0 copies per µl (Fig. 1). This corresponds well with the last amplification curve that crossed the threshold for detection, which should contain ~ 5 copies of the fusion gene.

### Screening of vestibular schwannoma samples for the presence of the SH3PXD2A-HTRA1 fusion transcript

**Fig. 2:**
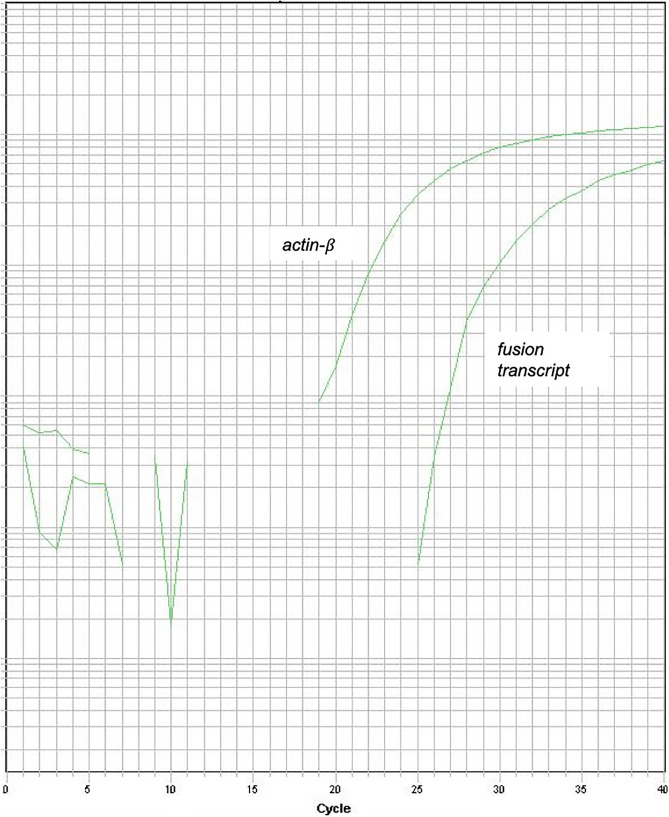
Detection of the fusion transcript using real-time PCR. The amplification plot for the actin-β (Ct:21.6) (internal control) and the fusion transcript (Ct:28.5) is shown. Comparison with the dilution series showed that the amplification plot for the fusion corresponded well with the 1/100,000,000 dilution, which was estimated to contain approximately 470 copies

121 VS samples were screened for the presence of the *SH3PXD2A-HTRA1* fusion transcript, which was detected in one patient (Fig. 2). The patient was a 16-year-old female with a large cystic tumor, measuring 4.5 cm in largest diameter. RNAseq and WGS data for this tumor demonstrated the fusion breakpoint in intron 6 of *SH3PXD2A* and intron 1 of *HTRA1 (results not shown)*, which was also found in 10/12 tumors reported by Agnihotri et al. We did not detect mutations in the *NF2* gene in our WGS data of the fusion-positive tumor.

## Discussion

When attempting to replicate a previous study, it is crucial that the conditions are as similar as possible. We therefore synthesized (in vitro) a fusion gene that could serve this function. Indeed, this synthetic positive control was detected by the originally reported real-time PCR assay, which was able to detect as low as ~ 5 copies of the starting material. We were also able to detect the synthesized fusion in ethidium bromide-stained 2% agarose gel using the described primers *(results not shown),* thus verifying the function of our synthetic fusion transcript as a positive control. However, although the real-time PCR assay was proven to be a sensitive test, we only detected the fusion in a single patient. This is in strong contrast to the results presented by Agnihotri et al., who reported that 11% of their VS patients were positive for the fusion transcript. Our study implies that the *SH3PXD2A-HTRA1* fusion is a rare event in vestibular schwannoma.

Both in vitro and in vivo data supported upregulation of pMEK-pERK in patients positive for the fusion transcript. Given the therapeutic potential of MEK-inhibitors in treating *SH3PXD2A-HTRA1* fusion-positive schwannomas, it is important to establish these tumors as a true subset of schwannomas. Although our data would suggest that the fusion is a very rare event, our material might represent an extreme. This underlines the importance of other research groups to investigate whether the fusion is also present in their cohorts of VS patients.

## Supplementary Information

Below is the link to the electronic supplementary material.

Supplementary file1 (EPS 187 kb) Schematic overview of the synthesis of the SH3PXD2A-HTRA1 fusion transcript.

Supplementary file2 (PDF 108 kb)
