# Supplementary material for: The SH3PXD2A-HTRA1 fusion transcript is extremely rare in Norwegian sporadic vestibular schwannoma patients
Source: J Neurooncol. 2021 Jul 2;154(1):35–40. doi: 10.1007/s11060-021-03796-6 (PMC8367919; doi:10.1007/s11060-021-03796-6)
Supplement: Supplementary file 2 — Supplementary file2 (PDF 108 kb) [file 11060_2021_3796_MOESM1_ESM.pdf]

Supplementary material:

Supplementary table 1. Primer information

Oligo name	Sequence
SH3XPD2Aex4F	TGAAGAGACTGAAGCCCATC
Fusion_R	GTTGGGATCTTCCTGCCCTGATTTCCTCTTGGAAC
Fusion_F	GTTCCAAGAGGAAATCAGGGCAGGAAGATCCCAAC
HTRA1R_HUS	CGACACAATAAACCCAGACC

Supplementary table 2. PCR setup, amplifying the HTRA1 and SH3PXD2A genes

Reactions	Primers	PCR product (bp)
SH3PXD2A amplification	SH3XPD2Aex4F	173
	Fusion_R	
HTRA1 amplification	Fusion_F	176
	HTRA1R_HUS	

94°C	10m	
94°C	30s	x 35
58°C	30s	x 35
72°C	30s	x 35
72°C	7m	
4°C	∞	

Supplementary table 3. PCR setup for amplification of the HTRA1-SH3PXD2A fusion transcript

SH3PXD2A-HTRA1 fusion amplification	*Template*:	*SH3PXD2A + HTRA1*	
	Primers:	SH3PXD2Aex4F (forward)	HTRA1R_HUS (reverse)

2 μl of the product from each of the previous reactions (for HTRA1 and SH3PXD2A, respectively) were mixed and used as DNA template in a new PCR reaction, yielding the fusion transcript. This reaction first ran 15 cycles without primers, which allowed annealing of the extension overlaps. The annealing temperature was 65° C, but the other parameters

were the same as before. After addition of 2 μl of each of the primers, the reaction ran an additional 30 cycles with an annealing temperature of 58° C. See supplementary figure for a schematic overview of the process.

a) *The reaction first ran 15 cycles without primers, enabling annealing of the extension overlaps.*

94°C	10m
94°C	30s
65°C	30s
72°C	30s
72°C	7m
4°C	∞

x 15

b) After addition of 2 μl of the PCR primers, the setup ran an additional 30 cycles.

94°C	10m
94°C	30s
58°C	30s
72°C	30s
72°C	7m
4°C	∞

x 30

Supplementary table 4. Primers and probe, real-time PCR

Forward	*GGCAGTTCCAAGAGGAAATCAG*
Reverse	*CCACGTCCGCGATAAAGTTATATT*
Probe	*ATGGCGCAAACTGTTGGGATCTTC*
